# Inquiries and Objections in Regard to Some Parts of a Communication from Dr. Henry on the Treatment of Fevers and Inflammations without Mercury

**Published:** 1847-08

**Authors:** Uri P. Golliday

**Affiliations:** Kickapootown, Illinois


### ARTICLE V.

*Inquiries and Objections in regard to some parts of a communication from Dr. Henry, on the Treatment of Fevers and Inflammations without Mercury.* By Dr. Uri P. Golliday, of Kickapootown, Illinois.

In the last number of the last volume of the Journal there is a communication from Dr. Henry on the treatment of fevers and inflammation without mercury, in which opium, in doses of four to six grains is highly recommended, not only in fevers but "in the treatment of all acute pnenumonic affections."

Although the opiate treatment of various modifications of febrile and inflammatory disease can scarcely be said to be a new one, as notices of its value have occasionally been brought before the profession for several years past; yet, in the *doses* (four to six grains) spoken of, there may be but little doubt of its originating with Dr. Henry.

Speaking of the treatment of coryza, Dr. Christison says "for twenty years I have been accustomed to see it stopped at once by a full opiate given on the first day of its appearance—during the same period I have often seen a common catarrh without fever, cut short in like manner, if taken on the first, second or perhaps even the third day." The doctor also names *Febrile Catarrh*, (meaning, I suppose, mild acute bronchitis,) *Epidemic Influenza*, and acute internal inflammations, with some explanatory modifications in the same category. (See Edinburgh Month. Jour. of Med. Sci., February, 1841, as quoted by Brathwaite's Retrospect, part 3, p. 45.)

Dr. Sobernheim, also gives us a summary of the diseases in which he found opium beneficial, and names intermittent fevers when offering a nervous character; inflammations of membranous, glandular, and sensitive organs; rheumatism and gout; catarrhal complaints, &c., &c.; but with the opium he combines antimony and mercury in the treatment of catarrhal diseases. (Rankin's Abstract, as quoted by Western Journal, December, 1845.) Professor Williams, of the University College, London, recommends it in doses of two or three grains of the aqueous extract, or from thirty to sixty minims of the liq. opii sedativus, or of Squire's tincture of the bi-meconate of morphia. But he says this plan can only be adopted where the bleeding has been sufficient to affect the action of the heart, almost, if not quite, amounting to syncope. (See Dis. of Respiratory system, Ed., 1845, p. 297.)

From these quotations (and their numbers might be very easily increased) it appears evident that the general plan is a good one, and whilst we have heretofore, and expect hereafter, to adopt it in all cases of fever where it is not contra-indicated by a tendency to disease of the brain, yet, with all deference to Dr. Henry, we would wish a little more light on the subject before we would unhesitatingly adopt it "in the treatment of *all* acute pneumonic affections."

The doctor very modestly observes—" When the practice receives the partial sanction of such names as Stokes, Bell, and Griffin, I shall expect that the suggestions of an obscure country practitoner will secure from the profession a sufficient degree of consideration to induce a trial of the remedy." But, notwithstanding this array of great names, there are some "philosophical theories" that interpose so as to prevent a too hasty adoption of the plan in all cases. Although I am of opinion that nosological distinctions and definitions have done much to impede the progress of practical medicine, and that an everlasting theorising in reference to the therapeutical application of remedies, may only "darken counsel by words without knowledge," yet I am persuaded we canot wholly divest our minds of "theories" in the application of remedies to the cure of disease, especially when the physiological effect of any article in question may have been previously ascertained with some considerable degree of certainty. It is worthy of remark, that those diseases in which the opiate plan of treatment is most highly recommended, are those involving the nervous system more or less, and, as Sobenheim expresses it, of the membranous, glandular, and sensitive organs; and whilst we might include intermittent fever, coryza, dysentery, pertussis, &c., in this catenation, we might at the same time be disposed to exclude diseases of the brain, those involving the parenchymatous structure of the lungs, and all others where there was a tendency to congestion of blood.

Pareira says—" From the effects of opium on the cerebrospinal system the following inferences may be drawn:

"1. That it is an objectionable agent in apoplexy, phrenitis, and paralysis.

"2. That under proper regulations it is a remedy which may be used to stimulate the cerebro vascular system," &c., &c.

" A knowledge of the effects of opium on the organs of respiration leads to the following conclusions:

"1. That this agent is contra-indicated in difficulty of breathing, arising from a deficient supply of nervous energy as in apoplectic cases; that it is improper where the venous is imperfectly converted into arterial blood; and, lastly, that it is improper in the first stage of catarrh and peripneumony, both from its checking secretion, and from its influence over the process of arterialization." (Materia Medica, pp. 697, 699, vol. ii.)

From some experiments made on animals by W. H. Judd, Esq., and abridged from the Med. Bot. Transactions by Braithwaite's Retrospect, part i, p. 58, the following " comparative view of the effects and changes after conium and of those after opium" is derived:

| CONIUM. | OPIUM. |
| --- | --- |
| Brain unnaturally free from blood; ventricles almost dry. | Brain gorged by blood; ventricles full of serum. |
| Lungs empty of blood. | Lungs so full of blood that it runs out in a stream on cutting them. |
| Stomach, its villous coat and œsophagus white. | Villous coat and œsophagus red. |
| Right side of the heart gorged by dark blood. Left empty. | Heart's cavities all containing blood. |

This is but a part of the comparative view, but enough for our present purpose; the reader is referred to the citation above for the balance, and for some very interesting remarks of Mr. Judd.

From these experiments we learn that the brain and lungs are full—are gorged with blood, from the use of opium. That such a state of these organs obtains more or less in man when a full dose of opium is taken, may be easily inferred from the fact that, with a more laborious respiration, there is a sense of fulness and tension in the chest and in the head; and in many cases there is increased redness of the face, with a swollen and turgid appearance of the vessels—in a word, there appears to be an excitation of the whole cerebro-vascular system.

As said above, we cannot always wholly divest our minds of "theories" in the application of medicines to the cure of disease; to do so in the present state of medical knowledge, would be to give medicine without reference to the pathological condition of the patient, or the physiological effects of remedies, an empiricism so gross that no man of feeling or honor could engage in it.

In pneumonia, the inflammation, engorgement and hepatization, in their different stages and modifications, constitute the disease or the results of a diseased action, which the physician wishes to remove. With this object in view, he would hesitate to use an article, proposed as the chief, or even as a very considerable part of the means of cure, or indeed in any other way than as a palliative under certain circumstances; when the experiments on animals, and the sensations produced in his own person by its use, make him believe it would cause at least one (and of course aggravate the others) of those very conditions constituting the diseased action he wished to remove.

These considerations form some of the reasons why, without further knowledge on the subject, I would fear "to give the practice a trial" "in all acute pneumonic inflammations."

---

ARTICLE VI.

